# The Growth of Reform

**Published:** 1905-08-05

**Authors:** 


### HOSPITAL ADMINISTRATION.

### CONSTRUCTION AND ECONOMICS.

### THE GROWTH OF REFORM.

### THE ROYAL SOUTH HANTS AND SOUTHAMPTON HOSPITAL.

(*Continued from page* 316.)

TWELVE YEARS' IMPROVEMENTS AND CHANGES.

By Miss M. Mollett.

II. What the County Hospital has become in 1905.

When the committee finally agreed to rebuild and reorganise the hospital, it was fortunately decided to treat the institution as a whole, and not to confine the improvements to any one department. Consequently, there is not one branch that has not been altered, enlarged, and brought into line with modern requirements, and that in a most generous and thorough manner. The old hospital has been practically rebuilt and brought up to date—new wards, laundry, theatre, mortuary, nurses' home, all of the newest type, have been added; the medical staff, both visiting and resident, has been increased; the nursing staff has been very largely increased and the training school developed, the diets have been improved and many domestic alterations have been introduced which, though trivial when enumerated individually, count enormously towards comfort and efficiency when added together.

For the building alterations the committee called in an expert firm of hospital architects, Messrs. Young and Hall, and under Mr. Hall's superintendence the whole building has been remodelled and rebuilt.

They seized upon the old building and tore down staircases and pulled down walls, broke fresh windows in the wards that were insufficiently ventilated, adding bathrooms and lavatories on the newest principles, lined throughout with glazed bricks and fitted with modern sluice pans and sinks. They gave us proper passages by which to enter our reorganised wards, they swept away the small wards, and converted them into offices, board-rooms and waiting-rooms, and did away for ever with the little operating-room under the roof. The whole of the hospital, front and back, was fresh cemented, and we lived and worked throughout the upheaval, never closing a bed more than could actually be helped, and yet I am alive to tell the tale. For years I never went to bed without shaking my pillow to remove the grit, for everything was smothered in fine dust, whilst I woke up every morning about five to hear the workmen thundering on iron girders or pecking away at stubborn stones that had to come down.

The very first new thing we built was a laundry—a fine new steam laundry—with sorting-room, engine-room, wash-house, ironing-room, etc., all complete and capable of coping with all the washing of the hospital; and last, but not least, provided with a full complement of hands to work the same. Where the old staircase, opposite the front entrance, used to wind, now stretches a handsome corridor lined with glazed brick and floored with terrazzo, leading to the "new block." And the first part of the new block to be built was the theatre, which stands well into the garden, built on arches with no basement.

It is a beautiful building with double swing doors separating it from the corridor, and it contains a theatre with excellent north light, surgeon's room, anæsthetic room, and recovery room, all lined with opalite and floored with terrazzo and heated with copper hot-water coils. I need hardly say that it is fitted throughout with every modern surgical necessary and plenty of hot water. Of course, all the new block is lighted by electricity, which is also used for all the boiling and sterilising in the theatre. At the end of the corridor are the two new wards which are the pride of the establishment; a block of two surgical wards for men and women, complete to the smallest detail. There are 25 beds in each; they are 100 feet long and are magnificently ventilated. They have large balconies facing south, protected by awnings in the summer; the floors are constructed of steel and concrete faced with marble terrazzo, whilst the highly glazed walls can be easily washed and polished like windows. These wards are also built on arches, and are free from basements of any kind.

The mortuary, which alone cost over £1,000, is built in the same thorough manner, lined with glazed bricks, and floored with terrazzo, with a mortuary proper, a post-mortem room, a small room for cutting sections, etc., and a room in which the friends can see their dead. A small infectious cottage, very bright and convenient, was also added, properly furnished and equipped. The out-patient department, which was built in 1887, separate from the hospital proper, is still in use; but we have added to the main building a two-roomed casualty block for night and accident work, the surgery fitted much like a small theatre, and two years ago the committee placed the coping-stone on the work by building a new nurses' home, razing to the ground the insanitary cottages which had borne that name. The new home contains 44 single bedrooms—not those abominations, cubicles—three sitting-rooms, eight bathrooms, with an endless supply of hot water, boxrooms on every landing, hot-air room for drying skirts, etc., the whole building heated by hot-water coils. When I add that the whole of these buildings and alterations were not only completed but paid for in eight years, and that there is now not one farthing owing for building, I think you will allow that it is a great achievement for a county town of the size of Southampton. When I stand in the garden and contemplate the noble line of buildings that form the hospital, I feel a pardonable glow of vanity in being the matron of such an imposing structure.

The Nursing Staff.

But it is of little use having a fine building if you have not the necessary staff to work it. More than one county hospital matron has complained bitterly to me of having to make bricks without straw, to nurse and clean a large hospital on an all too meagre allowance of hands. I must say my experience has been that the committee has always been most ready to sanction all needful increase both in the nursing and domestic staff—of course, not at once, but gradually.

The original scheme for the training of probationers—started before I came—was based on the, from my point of view, altogether wrong principle, of a two-years' training with certificate, no salary being given. Also to train and certificate one-year probationers at £30. Neither of these arrangements worked well. In 1896 we added one year to our training, with payment for the third year; in 1899 we abolished one year's training entirely, and arranged to pay our probationers a small salary from the beginning of their training.

But the greatest difficulty at first was to get probationers at all. The hospital was not well known, and it had not even locally a good reputation as a training school, and last, but not least, it offered no salary. The small number of nurses made the work desperately hard, and I have often had to call up a probationer in the night who had been on duty all day before, whilst the lack of servants made it necessary to use the probationers very much as charwomen. It has taken time and more hard work than people would believe to build up what is now a really very good county training-school. We have 30 probationers with seven day-sisters, the assistant matron, and a night superintendent. Their lectures are given by three of the visiting staff, with classes by the matron. They receive their certificates after passing their examinations, taking nursing notes of cases in the wards, and satisfying the matron as to their ward-work and general conduct. We have a Nurses' League for the certificated nurses of our hospital, which is managed by the nurses themselves, and we even contrive to print a bi-annual journal without any outside assistance. We are collecting a good nurses' library, whilst the nurses subscribe for their journals, and I have not the smallest difficulty in obtaining any amount of probationers I require.

Of course we have now an adequate staff of probationers on night duty. Resident wardmaids are attached to all the wards, and here again the supply exceeds the demand, and we pride ourselves on the years they stay; whilst a proper staff of maids, whose sole duty it is to attend to the Nurses' Home, make it possible to keep it really clean, though it is never spotless enough to satisfy my assistant matron. The servants are sufficient for all reasonable requirements, and the committee show a generous appreciation of the need for having windows cleaned, walls swept, and floors polished.

The equipment of the wards is excellent and sufficient, if not lavish; there are ample stores of all that is needful, and plenty of those little things that make hospital nursing so much easier now than it was 20 years ago.

The diets are not only more liberal, but greater attention is now paid to variety and the extras which, at small cost, make an enormous difference to the patients' meals.

It is impossible within the limits of an article to give a fuller description of all the changes that have taken place, but the above will give a fair idea of the progress and improvement of the last 12 years.

